# Operation for Removal of Post-Nasal Fibroid Tumor

**Published:** 1898-08

**Authors:** C. J. Sihler

**Affiliations:** Kansas City Veterinary College, Kansas City, Mo.


### REPORTS OF CASES.

### OPERATION FOR REMOVAL OF POST-NASAL FIBROID TUMOR.

By C. J. Sihler, V.S.,
KANSAS CITY VETERINARY COLLEGE, KANSAS CITY, MO.

The rarity of an operation involving the removal of a post-nasal or pharyngeal tumor through the trachea is a justification for the recital of a successful case.

A horse belonging to Mr. Culver, of Kansas City, Mo., and driven by him for some years as a family horse, became useless as such owing to an extreme difficulty in breathing. The animal was sent to a farm some distance from the city, with the expectation that rest would bring relief. After some weeks, no improvement being shown, the owner called in a local veterinarian who was at a loss to determine the cause of the trouble. Though of no particular money-value, the owner of the animal determined to continue his efforts for its relief, and requested Dr. R. C. Moore to examine the animal.

On a cursory examination it was found that the difficulty was caused by an obstruction of the nasal passages, the nature of which could not be determined with the facilities at hand. Upon suggestion of Dr. Moore, the animal was sent to the Kansas City Veterinary College Hospital for further investigation and surgical relief. The horse arrived apparently very much distressed, breathing with much difficulty, the nostrils discharging a thick, viscid fluid. Examination showed that the labored breathing was due to a dense growth of some kind in the superior part of the nasal chambers. A tracheotomy-tube was introduced temporarily, to afford relief for the time being. The animal was allowed to rest until the next day, when a more complete examination was made. By passing a rubber catheter up the left nostril as far as the posterior nares an obstruction was found, preventing its further passage. A like obstruction was found in the right chamber. By closing the tracheotomy-tube and right nostril the animal could, by considerable effort, breathe through the left nostril. When the left nostril was closed it was impossible for him to breathe, showing that the right chamber was entirely occluded. It was decided to endeavor to remove the growth, but how?

The obstruction was situated so far up the nasal passage and so completely filled both chambers that it could not be grasped and removed through the nasal passage, and to cut down through the frontal sinuses and effect its removal would require a very large opening, which when healed would disfigure the animal, thus rendering him worthless as a driving-horse. A trephine-button taken from the frontal sinuses would only determine the character of the growth and would be of no practical value. A wire loop was passed up the nostril with a view of passing it over part of the growth and removing it to determine its nature, but this was not possible, as the wire would not pass beyond the anterior surface of the growth. However, being somewhat assured of its non-malignant character by the absence of hemorrhage from the nostrils, it was finally decided to make an opening through the trachea and endeavor to determine the nature of the growth and, if possible, remove it through that channel.

All the instruments necessary were cleaned and placed on a tray, with cotton, sponges, and a 5 per cent. solution of creolin. The animal was cast and secured, and chloroform administered through the tracheotomy-tube. It required about one hour to place the horse completely under the influence of the anæsthetic, probably owing to the very slow respirations, which varied from four to six per minute. Then the temporary tube was removed and one with rubber tampon substituted, and chloroform continued. The hair was clipped from the intermaxillary space and neck as far down as the tracheotomy-tube, and the parts washed with a 5 per cent. solution of creolin. An incision was made through the skin and subcutaneous tissues, and hemorrhage checked by sponging, after which an opening was made through the first four rings of the trachea and the cricoid cartilage of the larynx. The small tracheal vessels were caught with artery forceps and occluded by torsion, and the trachea and exposed larynx cleaned with the creolin solution. The hand was then passed through the larynx and pharynx, with some force carefully applied. The growth was readily reached and found to be firmly attached to the base of the vomer and extended around the septum nasi, the ends extending into the nasal chambers. It was impossible to get an écraseur-chain through the opening and over the growth, and it could not be torn loose with the fingers. The operators were in a quandary how to proceed, when the farmers' friend, the baling-wire, was thought of, and a loop made of it was passed up the right nostril, the hand passed through the opening in the trachea, raising or depressing the growth as occasion required to let the wire pass. This loop was drawn through the opening in the trachea and enlarged sufficient to pass over the growth. Force was now applied and the growth torn from the base of attachment, and removed through the nostril. In its passage it probably compressed the turbinated bones, but apparently did not injure them. The growth proved to be a fibroid polypus, eight inches in length and three inches in diameter, the largest of its kind ever observed by the operators. Its attachment was very broad, about five inches in length and two and one-half inches in width. The parts were sponged with the creolin solution and the posterior nares, pharynx, and larynx packed with cotton, and a suture through the external wound was taken.

This concluded what the operators deemed an unusual operation. The animal rallied nicely, and in about an hour was walking about. The following day the cotton was removed from the pharynx and larynx, that which filled the posterior nares being allowed to remain for forty-eight hours. It was found necessary to cast the horse to remove it, as the animal would not allow the dressing forceps to be introduced into the wound. The tracheotomy-tube was removed the second day and the parts were cleaned daily with the creolin solution. The wound healed kindly, and the fourth week it had entirely closed and the animal was pronounced well.

